# Testing for Phylogenetic Signal in Single-Cell RNA-Seq Data

**DOI:** 10.1089/cmb.2022.0357

**Published:** 2023-04-18

**Authors:** Jiří C. Moravec, Robert Lanfear, David L. Spector, Sarah D. Diermeier, Alex Gavryushkin

**Affiliations:** ^1^ Department of Computer Science, University of Otago, Dunedin, New Zealand.; ^2^ School of Mathematics and Statistics, University of Canterbury, Christchurch, New Zealand.; ^3^ Division of Ecology and Evolution, Research School of Biology, Australian National University, Canberra, Australia.; ^4^ Cold Spring Harbor Laboratory, New York, New York, USA.; ^5^ Department of Biochemistry, University of Otago, Dunedin, New Zealand.

**Keywords:** cancer, phylogenetics, RNA-seq, single cell

## Abstract

Phylogenetic methods are emerging as a useful tool to understand cancer evolutionary dynamics, including tumor structure, heterogeneity, and progression. Most currently used approaches utilize either bulk whole genome sequencing or single-cell DNA sequencing and are based on calling copy number alterations and single nucleotide variants (SNVs). Single-cell RNA sequencing (scRNA-seq) is commonly applied to explore differential gene expression of cancer cells throughout tumor progression. The method exacerbates the single-cell sequencing problem of low yield per cell with uneven expression levels. This accounts for low and uneven sequencing coverage and makes SNV detection and phylogenetic analysis challenging. In this article, we demonstrate for the first time that scRNA-seq data contain sufficient evolutionary signal and can also be utilized in phylogenetic analyses. We explore and compare results of such analyses based on both expression levels and SNVs called from scRNA-seq data. Both techniques are shown to be useful for reconstructing phylogenetic relationships between cells, reflecting the clonal composition of a tumor. Both standardized expression values and SNVs appear to be equally capable of reconstructing a similar pattern of phylogenetic relationship. This pattern is stable even when phylogenetic uncertainty is taken in account. Our results open up a new direction of somatic phylogenetics based on scRNA-seq data. Further research is required to refine and improve these approaches to capture the full picture of somatic evolutionary dynamics in cancer.

## 1. INTRODUCTION

Phylogenetic analysis is an approach that relies on reconstructing evolutionary relationships between organisms to determine population genetics parameters such as population growth (Heled and Drummond, 2008; Kingman, 1982), structure (Müller et al, 2017a), or geographical distribution (Lemey et al, 2010, Lemey et al, 2009). Typically, the reconstructed phylogeny is not the end-goal. Using previously estimated trees, various evolutionary hypotheses can be explored, such as the evolutionary relationship of traits carried by individual taxa (Freckleton, 2012; Grafen and Hamilton, 1989; Pagel et al, 2004).

Within-organism cancer evolution is increasingly being studied using population genetics approaches, including phylogenetics (Alves et al, 2019; Alves et al, 2017; Caravagna et al, 2020; Caravagna et al, 2018; Detering et al, 2019; Kuipers et al, 2020; Malikic et al, 2019; Navin et al, 2011; Singer et al, 2018; Schwartz and Schäffer, 2017; Werner et al, 2020; Yuan et al, 2015), to understand evolutionary dynamics of cancer cell populations. These approaches have shown promise to be developed into therapeutic applications in the personalized medicine framework (Abbosh et al, 2017; Gerlinger et al, 2012; Rao et al, 2020b).

Specifically, the clonal composition of tumors, metastasis initiation, development, and timing can be reconstructed using phylogenetic methods (Angelova et al, 2018; Alves et al, 2019; El-Kebir et al, 2018; Yuan et al, 2015). Unlike other evolutionary processes prone to events such as hybridization or horizontal gene transfer, population dynamics of somatic cells is underpinned by a strictly bifurcating clonal process driven by cell division. This is in perfect agreement with theoretical assumptions routinely applied in stochastic phylogenetic models such as coalescent (Hudson^1990^; Kingman, 1982; Posada, 2020) or birth-death processes (Aldous, 2001; Aldous, 1996; Komarova, 2006).

From the methodological perspective, however, cancer is an evolutionary process with unique characteristics, which are not modeled in conventional phylogenetic approaches. These include a high level of genomic instability with structural changes (gene losses and duplications), which accumulate along with point mutations during the course of growth and evolution (Beerenwinkel et al, 2015; Posada, 2015).

Traditional whole genome sequencing (WGS) methods have been instrumental in understanding cancer mutational profiles and oncogene detection (Mardis and Wilson, 2009; Nakagawa and Fujita, 2018). DNA from a tissue sample is isolated and sequenced “in bulk.” This increases the total amount of DNA that improves coverage and reduces amplification errors. To establish the presence or absence of mutations, a variant allele frequency (VAF) is calculated and compared to a threshold, typically 10−20% (Strom, 2016). This filters out rare mutations present only in a few reads that are likely to be false positives or sequencing errors (Petrackova et al, 2019). More recently, bulk sequencing is used to study cancer evolution using phylogenetic methods, either by comparing VAF (Ling et al, 2015; Zhao et al, 2016; Zhai et al, 2017) or estimating copy number variants (CNVs) (Desper et al, 1999; Demeulemeester et al, 2016; Tarabichi et al, 2021).

However, the usage of bulk sequencing in this context is problematic. Bulk samples contain cells from multiple cell lineages, including non-tumor cells, such as immune or blood vessel cells (Racle et al, 2017), and there is strong evidence for a constant migration of metastatic cells between tumors (Aguirre-Ghiso, 2010; Cheung and Ewald, 2016; Casasent et al, 2018; Reiter et al, 2017). High VAF thresholds ignore tumor heterogeneity, but by lowering the threshold, mutations in nontumor cells or clonal lineages are retained instead. Sequences or mutational profiles derived from bulk samples thus have a chimeric origin (Alves et al, 2017).

A typical assumption in classical phylogenetics is that the sequences or mutational profiles represent individual taxonomic units, either individuals or populations of closely related individuals. If these methods are used on the data from bulk samples, the reconstructed trees are not phylogenies describing an evolutionary history, but evolutionarily meaningless sample similarity trees (Alves et al, 2017). To address this issue, phylogenetic trees are reconstructed by estimating the sequential order of somatic mutations using VAF from one or multiple tumor samples (Deshwar et al, 2015; El-Kebir et al, 2018; Miura et al, 2018). Given the tumor heterogeneity and insufficient read depth to reliably estimate VAF, this is not a simple problem and the performance of current methods is limited (Miura et al, 2020).

Single-cell DNA sequencing (scDNA-seq) does not suffer from the chimeric DNA origin of bulk sequencing as each DNA segment is barcoded to guarantee its known cell of origin. Recent progress in WGS technology made sequencing individual cells cost-efficient (Gawad et al, 2016) and this approach is now regularly used for the phylogenetic reconstruction of metastatic cancer or the subclonal structure of a single tumor (Leung et al, 2017; Myers et al, 2019; Potter et al, 2013; Roth et al, 2016). However, this increased resolution comes with additional complications. Current methods are not sensitive enough to sequence DNA from a single cell and DNA amplification is required (Gawad et al, 2016). This process suffers from a random bias with different parts of the genome amplified in different quantities or not at all (Satas and Raphael, 2018).

In addition, polymerase does not replicate DNA without error; this can have a significant impact if the replication errors occur early in the amplification process (Gawad et al, 2016). This does not only increase the error rate for identified single nucleotide variants (SNVs) but also a large proportion of SNVs might be simply missing (Hicks et al, 2018). The advantages associated with scDNA-seq led to the development of novel approaches that tackle these challenges using an error model to correct for amplification errors and false-positive SNV calls (Luquette et al, 2019; Kozlov et al, 2022; Zafar et al, 2019).

Similar technological development led to proliferation of single-cell RNA sequencing (scRNA-seq), which, compared to traditional bulk RNA sequencing, enabled detection of gene expression profiles for individual cells in the tissue sample (González-Silva et al, 2020; Jerby-Arnon et al, 2018; Olsen and Baryawno, 2018; Müller et al, 2017b). This allows understanding tumor heterogeneity by identifying different cell populations (Andrews and Hemberg, 2018), estimating immune cell content within a tumor (Yu et al, 2019), or even identifying individual clones and subclones, as they can differ in their behavior (Fan et al, 2020).

However, as the levels of RNA expression vary between genes and cells, the amplification problems of scDNA-seq that cause unequal expression and dropout effects are more pronounced in scRNA-seq. There is an increased interest for SNV calling on scRNA-seq data using bulk SNV callers (Chen et al, 2016; Liu et al, 2019; Poirion et al, 2018; Schnepp et al, 2019) and specialized CNV callers (Kuipers et al, 2020; Harmanci et al., 2020a,b; Gao et al, 2021) as this allows for identification of mutations in actively expressed genes.

In this work, we test if expression values and SNVs inferred from scRNA-seq contain phylogenetic information to reconstruct a population history of cancer. We reconstruct phylogenetic trees using expression values and SNVs from three different datasets. We then compare and test these phylogenies against expected evolutionary history to determine whether scRNA-seq data contain phylogenetic signal.

## 2. METHODS

### 2.1. Experimental design

To test if scRNA-seq data contain phylogenetic signal, we select datasets with multiple regional samples and test if cells from a regional sample are phylogenetically closer to each other than to cells from other samples.

Methods that reconstruct phylogenies from sequence data assume that the data were generated using an evolutionary process. Under this assumption, an effect of a low phylogenetic signal would produce a high mutation rate and/or star tree-like structure. However, both high mutation rate and star trees are biologically plausible in case of rapid population expansion, such as during a rapid metastatic spread (Schwarz et al, 2015). Classical methods for estimation of phylogenetic signal, such as Pagel's λ (Pagel, 1999) or Blomberg's κ (Blomberg et al, 2003) (see Münkemüller et al (2012) for review), are not applicable, as they test the presence of a phylogenetic signal of an observed trait based on known phylogeny.

An alternative method to test whether the data do contain a phylogenetic signal that we employ in this article is to compare the resulting phylogeny to an expected relationship, such as the expected monophyly of groups of taxa obtained from multiple regional samples, for example, phylogenetic clusters of cells from healthy tissues or individual metastases. To further account for migration and uncertainty, we do not require cells from a single regional sample to be monophyletic, but test whether cells from a regional sample are phylogenetically closer to each other than to cells from other samples using phylogenetic clustering tests.

### 2.2. Dataset selection

We perform our phylogenetic analyses on three different datasets, a new unique molecular identifier (UMI)-based dataset of breast cancer-derived xenografts (BCX), and two previously published datasets, a UMI-based dataset of small intestinal neuroendocrine cancer (INC) (Rao et al, 2020a) and a non-UMI-based dataset of gastric cancer (GC) (Wang et al, 2021). These datasets contain primary and metastatic cells from multiple regional samples, allowing us to assess the performance of the phylogenetic analysis using the phylogenetic clustering tests. We expect that primary, metastatic, and cells from regional samples will each cluster together, forming cell-type and region-specific clades.

The BCX dataset consisted of three individual specific tumor samples T1, T2, and T3, seeded from the same population of cells, and two matched circulating tumor cell (CTC) samples CTC1 and CTC2. Each tumor was derived from the same population and shares a common ancestor; each tumor thus represents a distinct regional sample. We expected that cells isolated from each individual and cells for each sample form clusters. As the cell lineage MDA-MB-231-LM2 is highly metastatic (Minn et al, 2005), we do not expect CTC to form a single monophyletic clade, but a larger number of smaller clades embedded inside the tumor cells of a sampled individual.

The INC dataset from Rao et al (2020a) consisted of a primary tumor and a paired liver metastatic sample. Both samples contained a mixture of cancerous and noncancerous cells (fibroblasts, endothelial cells, and immune cells). We expect cancerous cells to form a cluster, with metastatic cells forming distinct clusters among the cancer cells.

The GC dataset from Wang et al (2021) consisted of 94 cells from a primary tumor and a lymph node of three patients (GC1, GC2, and GC3). We expect that for each patient, the lymph node cells would form a monophyletic lineage derived from the primary tumor cell, but due to the small number of cells, clustering of the primary tumor cells is also interpreted as a success.

### 2.3. Preparation of the BCX dataset

MDA-MB-231-LM2 (green fluorescent protein: GFP+) (Minn et al, 2005) cells were injected into the R4 mammary fat pad of Nu/J mice (250,000 cells per mouse, 3 mice), and tumor growth was monitored for 8 weeks. Mice were euthanized when tumor size approached the endpoint (2 cm). Tumors were resected and dissociated into single cells. To extract CTC, up to 1 mL of blood was drawn immediately posteuthanasia using cardiac puncture. Red blood cells were removed using RBC lysis buffer. All cells (tumor derived and CTC) were stained with 4′,6-diamidino-2-phenylindole (DAPI) and sorted for DAPI and GFP using a BD FACSAria cell sorter. Libraries were generated using the 10 × Chromium single cell gene expression system immediately after cell sorting, and sequenced on an Illumina NextSeq platform together to eliminate batch effect.

### 2.4. Mapping and demultiplexing

The BCX and INC reads were mapped with the Cellranger v5.0 software to the GRCh38 v15 from the Genome Reference Consortium using the analysis-ready assembly without alternative locus scaffolds (no_alt_analysis_set) and associated GTF annotation file. The Cellranger software performs mapping, demultiplexing, cell detection, and gene quantification for the 10 × Genomics scRNA-seq data.

Reads for the non-UMI GC dataset were mapped with the STAR v2.7.9a (Dobin et al, 2013), using the same reference and GTF annotation file.

### 2.5. Postprocessing expression data

For the INC and BC datasets, previously published expression datasets from Rao et al (2020a) (GSE140312) and Wang et al (2021) (GSE158631) were used. For the BCX dataset, we have used the filtered expression values produced by the Cellranger.

#### 2.5.1. Standardizing expression values

The filtered feature-barcode expression values from Cellranger were processed using the R Seurat v4.0.4 package (Stuart et al, 2019). Expression values from different regional samples (or individuals in case of BCX) were merged and analyzed together. The expression values for each gene were centered (μ=0) and rescaled ($\sigma^{2}=1$). No normalization or filtering was done at this step.

#### 2.5.2. Discretizing expression values

To reconstruct phylogenies, expression values need to be discretized as phylogenetic software does not support tree reconstruction from continuous data. The rescaled expression values were categorized into a five-level ordinal scale ranging from 1 (low level of expression) to 5 (high level of expression). The five-level scale was chosen to capture the data distribution of rescaled expression values and represent a compromise between introducing data noise with too many levels or artificial similarity with only a few categories.

Interval ranges, according to which the values were categorized, were chosen according to the 60% and 90% highest density intervals (HDI), the shortest intervals containing 60% or 90% of values, respectively. The values inside the 60% HDI were categorized as normal, values inside the 90% HDI, but outside the 60% as increased/decreased expression, and values outside the 90% HDI as an extremely increased/decreased expression.

Genes that contain only a single categorized value for each cell were removed as phylogenetically irrelevant and the discretized values were then transformed into fasta format.

#### 2.5.3. Recording unexpressed genes as unknown data

The amount of coverage in a standard bulk RNA-seq expression analysis is usually sufficient to conclude that genes for which no molecule was detected are not expressed (Lähnemann et al, 2020). In scRNA-seq, however, the sequencing coverage is very small, dropout effect is likely, and thus this assumption does not hold. This is especially a problem for non-UMI-based technologies (Cao et al, 2021), but not entirely absent from the UMI-based technologies as well due to biological and technological processes (Hsiao et al, 2020; Townes and Irizarry, 2020).

According to the standard expression pipeline, these values are commonly treated as biological zeros, that is, no detected expression of a particular gene, and have a significant impact on the data distribution during the normalization and rescaling steps (Hicks et al, 2018; Townes and Irizarry, 2020). Without an explicit model of dropout effect to account for technical or biological variation, these values might be more accurately represented as unknown values rather than true biological zeros (Van den Berge et al, 2018). We have modified the Seurat code to treat these values as unknown values (NA in R) and included modified functions in the phyloRNA package.

We will further use *data density* to describe the number of unknown values in both expression and SNV datasets, with 100% representing data set without unknown values, while 0% would represent a dataset formed entirely of unknown values.

### 2.6. SNV

#### 2.6.1. Preprocessing reads for SNV detection

The BAM files from Cellranger were processed using the Broad Institute's Genome Analysis ToolKit (GATK) v4.2.3.0 (Poplin et al, 2018) according to GATK best practices of somatic short variant discovery. Reads were sorted, processed, and recalibrated using GATK's SortSam, SplitNCigarReads, and Recalibrate.

#### 2.6.2. SNV detection and filtering

To obtain SNVs for individual cells of the scRNA-seq data, first, a list of SNVs was obtained by running Mutect2 (Benjamin et al, 2019), treating the data set as a pseudo-bulk sample, and retaining only the SNVs that passed all filters.

For the BCX dataset, Mutect2 was run in the tumor with matched normal sample using the parental cell linage MDA-MB-231 from Kidwell et al (2021) and Panel of Normals derived from the same source, see Supplementary Materials S1 for details.

For the IND and GC datasets, we have used Mutect2 in a tumor-only mode using the Panel of Normals and the GNOMAD germline data from the GATK best practice resource bundle.

SNVs for individual cells were then obtained by individually summarizing reads belonging to each single cell at the positions of the SNVs obtained beforehand using the pysam library, which is built on htslib (Li et al, 2009). The most common base for every cell and every position was retained, and base heterogeneity and CNVs were ignored. This SNV table was then transformed into fasta format.

### 2.7. Finding a well-represented subset of data

When treating the potentially unexpressed genes as unknown values, only a small proportion of the expression count values was known, with the data set derived from SNV suffering from the same problem due to the low number of reads for each cell.

While model-based phylogenetic methods can process missing data by treating the missing data as phylogenetically neutral, this significantly flattens the likelihood space, which can cause artifacts, convergence problems or increase computational time (Jiang et al, 2014; Wiens, 2006; Xi et al, 2016).

Published phylogenetic tools designed for single-cell DNA data sets ranged from 47 cells and 40 SNVs (Jahn et al, 2016) to 370 cells and 50 SNVs (Singer et al, 2018) or in an extreme case, 18 cells and 50,000 SNVs (Singer et al, 2018), with at most 58% of missing data across these data sets. In comparison, scRNA-seq technology can produce up to tens of thousands of cells with tens of thousand detected genes (Chen et al, 2019) and data reduction is often required.

To alleviate these issues, we employ two different filtering strategies to reduce the size of datasets, while preserving as much information as possible, a selection strategy, where a set of high-quality cells is selected, and a stepwise filtration algorithm, where a subset of data with the highest data density is selected. Under the selection filtering strategy, a set of cells is selected, either cells of interest from the expression analysis, or a fixed subset of cells with the highest data density. This allows for a construction of datasets of specific size.

The stepwise filtering algorithm aims to find a well-represented subset of the data. By iteratively cutting out cells and genes/SNVs with the smallest number of known values, we increase the data density until a local maximum or desired data density is reached. This is equivalent to the gene/cell quality filtering during the scRNA-seq postprocessing pipeline, such as in the Seurat's standard preprocessing workflow described above, where low-quality cells and genes are removed. The advantage of this method is that a desired density can be reached with the least amount of data removed.

### 2.8. Phylogenetic analysis

To reconstruct phylogenetic trees from the categorized expression values and identified SNVs, we used IQ-TREE v2.1.4 (Minh et al, 2020) and BEAST2 v2.6.3 (Bouckaert et al, 2019).

The IQ-TREE analysis was performed with an ordinal model and an ascertainment bias correction (-m ORDINAL+ASC) for the expression data, and a standard model selection was performed for the SNV data (-m TEST). Where the size of the dataset allowed, tree support was evaluated using the standard nonparametric bootstrap (Felsenstein, 1985) with 100 replicates (-b 100).

The BEAST2 analysis was performed with a birth-death tree prior (Kingman, 1982). Exponential population growth and BD model are not compatible; we have previously used the coalescent model (with exponential population growth) and forgot to (fully) update the text. For the expression data, the BEAST2 was run using ordinal model available in the Morph-Models package, while the SNV values were analyzed using the Generalized Time-Reversible model (Tavaré, 1986). For both the expression and the SNV data sets, BEAST2 was set to not ignore ambiguous states.

### 2.9. Phylogenetic clustering tests

To test if the phylogenetic methods were able to recover expected population history, we employ mean pairwise distance (MPD) (Webb, 2000) and mean nearest taxon distance (MNTD) (Webb, 2000). MPD is calculated as a mean distance between each pair of taxa from the same group, while MNTD is calculated as a mean distance to the nearest taxon from the same group. For each sample and samples isolated from a single individual, MPD and MNTD are calculated and compared to a null distribution obtained by permuting sample labels on a tree and calculating MPD and MNTD for these permutations. The *p*-value is then calculated as a rank of the observed MPD/MNTD in the null distribution normalized by the number of permutations.

The MPD and MNTD are calculated using the ses.mpd and ses.mntd functions implemented in the package picante (Kembel et al, 2010). For the Bayesian phylogenies, MPD and MNTD were calculated for a sample of 1000 trees from the posterior distribution and then summarized with mean and 95% confidence interval. For maximum likelihood phylogenies, MPD and MNTD were calculated from 100 trees, from the nonparametric bootstrap, and summarized in the same manner as Bayesian trees.

### 2.10. Code and data availability

Code required to replicate the data processing steps is available at https://github.com/bioDS/phyloRNAanalysis.

To aid in creating pipelines for phylogenetic analysis of scRNA-seq data, we have integrated a number of common tools in the R phyloRNA package, which is available at https://github.com/bioDS/phyloRNA.

Raw reads and expression matrices produced by Cellranger for the BCX dataset are in the NCBI GEO under the accession number GSE163210.

Alignments in the fasta format, reconstructed trees, and phylogenetic clustering test results are available at https://github.com/bioDS/phyloRNAanalysis/tree/processed_files.

## 3. RESULTS

### 3.1. Breast cancer-derived xenografts

#### 3.1.1. Sample overview

In total, five samples were used in this analysis, three tumor samples (T1, T2, and T3) and two CTC samples (CTC1 and CTC2). The number of cells isolated from the CTC3 sample was too small for scRNA sequencing and the sample was removed from the study. The number of detected cells in the tumor samples was generally smaller than in the CTC samples, but the reverse was true for the total number of detected UMIs—the number of unique mRNA transcripts (Table 1). In the T2 sample, a large number of cells but a small number of UMIs were detected in a similar pattern to the CTC samples.

**Table 1.** An Overview of the Breast Cancer-Derived Xenograft Dataset Used in This Work

						
*(a) Experiment overview*						
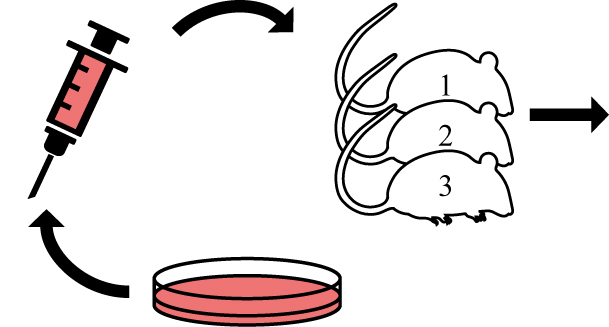						
*(b) Sample overview*						

Sample	Cells (FACS)	Cells (Cellranger)	Genes	UMI	UMI/cell	Data Density
T1	11,258	701	17k	3,167k	4,518	2.97%
T2	20,233	2,794	5k	69k	25	0.04%
T3	13,865	806	18k	2,876k	3,569	2.57%
CTC1	605	3,125	8k	129k	41	0.06%
CTC2	415	3,161	9k	155k	49	0.06%
Total	46,376	10,587	20k	6.4M	604	0.44%

In total, five samples were isolated from three individuals (a): three tumor samples (T1, T2, and T3) and two CTC samples (CTC1 and CTC2). For each sample, the number of cells from FACS, the number of identified by Cellranger, the number of detected genes, the number of UMIs, UMI/cell ratio, and the data density are reported (b).

CTC, circulating tumor cell; FACS, fluorescent-activated cell sorting; UMI, unique molecular identifier.

Compared to the fluorescent-activated cell sorting, Cellranger detected fewer cells for tumor samples, but more cells for the CTC samples. Cellranger classifies barcodes as cells based on the amount of UMI detected to distinguish real cells from a background noise (Lun et al, 2019). The large number of detected cells in the CTC samples is likely a result of lysed cells or cell-free RNA (Fleming et al, 2019). In all cases, the number of expression values across data sets was relatively low, with the best sample T3 amounting to about 3% of known expression values.

#### 3.1.2. SNV identification

To identify SNVs in scRNA-seq data, we first identified a list of SNVs by treating the single-cell reads as a pseudo-bulk sample. The total of 21,261 SNVs that passed all quality filters were identified this way. When these SNVs were called for each individual cell, the resulting data set had data density of <0.13%.

The expression data are expected to have higher data density than SNV because for expression quantification, a presence or absence of a molecule is sufficient, while for SNV, knowledge of each position is required. This expectation is confirmed in Table 1, where data density of the expression data is summarized. About 40% of 10,587 cells represented in this data set did not contain any positively identified SNV after filtering out false positives, these were relatively equally distributed among the T2 (1487), CTC1 (1379), and CTC2 (1324) samples. This represents a challenge from a data analysis perspective, given the large sample size and its small data density.

#### 3.1.3. Data reduction

With over 10,000 cells and more than 20,000 genes and SNVs, the unfiltered datasets would require substantial computational resources. An additional issue we have encountered in our data was a significant difference in the quality between individual samples, only five CTC1 and six CTC2 cells passed the quality filtering criteria of a minimum of 250 represented genes and a minimum of 500 UMI per cell, with no T2 cell passing the quality filtering. This contrasts with the T1 and T3 samples, where 701 and 806 passed the quality filtering criteria, respectively.

Due to this varied quality of samples, filtering data to a higher data density using the stepwise filtering algorithm leads to the removal of low-quality samples (T1, CTC1, and CTC2), which bars us from testing the phylogenetic structure using the phylogenetic clustering tests. For this reason, we have selected a small number of cells with the least amount of missing data from each sample using the selection filtering method. The small number of cells is not sufficient to represent the full diversity of the tumor, but allows us to test the phylogenetic relationship between individual samples without introducing a bias due to an unequal size of the samples.

A total of 58 cells were retained for both the expression and SNV datasets: 20 cells for T1 and T3 samples and six cells for T2, CTC1, and CTC2 samples. In these reduced datasets, genes that were not present in any of the cells or present only in a single cell are removed. The reduced expression data set contained 30% of known data distributed across 7,520 genes. The SNV data set contained 10% of known data distributed across 1,058 SNVs. These reduced data sets are analyzed using maximum likelihood and Bayesian method to further explore the topological uncertainty.

Reconstructed trees and phylogenetic tests for the data filtered to the 20%**,** 50%, and 90% data density using the stepwise filtering algorithm are provided in the Supplementary Materials S1.

#### 3.1.4. Phylogenetic reconstruction from expression data

**FIG. 1.**
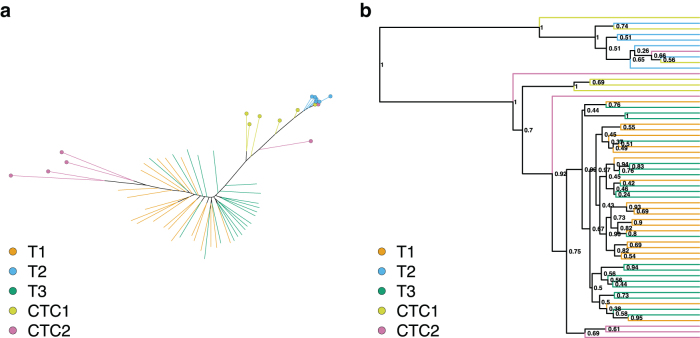
**(a)** Maximum likelihood and **(b)** Bayesian trees reconstructed from the expression data for the 58 selected cells. Terminal branches are colored according to cell's sample of origin (T1, T2, T3, CTC1, and CTC2). In the maximum likelihood tree, the T2, CTC1, and CTC2 samples are also marked with colored circles. For the Bayesian tree, Bayesian posterior values show the topology uncertainty. CTC, circulating tumor cell.

The maximum likelihood tree reconstructed from the reduced expression data set showed significant clustering of all samples (Fig. 1a). This is confirmed by the phylogenetic clustering tests where all, but CTC2 cells had a significant MPD *p*-value (Table 2). Four out of six CTC2 cells clustered together, but on the opposite side of the tree with phylogenetic proximity to the T1 cells. This close phylogenetic relationship suggests that T1 and CTC2 were isolated from a single individual. This pattern is further reinforced as T2 cells clustered in a single compact clade with phylogenetic proximity to the CTC1 sample. When this relationship was tested with phylogenetic clustering methods, both MPD and MNTD confirmed the strong clustering signal between T2 and CTC1. The same tests were not significant for the T1-CTC2 grouping, likely due to the presence of two nonclustering CTC2 cells.

**Table 2.** Test of Phylogenetic Clustering for the Reduced Dataset of the 58 Selected Cells

Groups	Cells	Expression (ML)		Expression (BI)		SNV (ML)		SNV (BI)	
		MPD	MNTD	MPD	MNTD	MPD	MNTD	MPD	MNTD
T1	20	^*^0.003	0.755	^*^0.001	^*^0.006	^*^0.001	^*^0.008	^*^0.001	0.434
T2	6	^*^0.001	^*^0.001	^*^0.001	^*^0.003	^*^0.001	^*^0.001	^*^0.001	^*^0.001
T3	20	^*^0.001	0.423	^*^0.001	^*^0.017	^*^0.002	0.660	^*^0.001	0.184
CTC1	6	^*^0.002	^*^0.004	0.992	0.479	^*^0.001	^*^0.001	^*^0.001	^*^0.002
CTC2	6	1.000	0.988	0.724	0.966	0.247	0.595	0.223	0.594
T1 and CTC1	26	0.059	0.407	0.139	0.261	0.475	^*^0.008	0.466	0.305
T2 and CTC2	12	0.997	0.027	0.970	0.165	0.998	0.800	0.977	0.317
T1 and CTC2	26	0.637	0.999	^*^0.005	0.932	^*^0.001	0.630	^*^0.001	0.943
T2 and CTC1	12	^*^0.001	^*^0.001	0.674	0.034	^*^0.001	^*^0.001	^*^0.001	^*^0.001

MPD and MNTD calculated for the ML and BI trees from the expression and SNV data. *p*-Values for MPD and MNTD were calculated for each sample (T1, T2, T3, CTC1, and CTC2) and expected clustering for cells isolated from a single individual (T1 with CTC1, and T2 with CTC2) and to test a possible mislabeling between CTC1 and CTC2 samples (T1 with CTC2, and T2 with CTC1). Significant *p*-values at α=0.05 after correcting for multiple comparisons using the False Discovery Rate method (Benjamini and Hochberg, 1995) are marked with an asterisk. Values of MNTD and MPD calculated for the ML bootstrap sample and BI posterior tree sample are available in the Supplementary Table S1.

^*^ Significant support.

BI, Bayesian; MNTD, mean nearest taxon distance; MPD, mean pairwise distance; ML, maximum likelihood; SNV, single nucleotide variant.

The phylogenies reconstructed from the same data using the Bayesian inference show a similar pattern of clustering (Fig. 1b, Table 2), although neither CTC1 nor CTC2 formed a compact cluster. The T2 and CTC1 connection is not supported, but about half the CTC1 cells were placed in a group with the T2 samples. Similar to the maximum likelihood tree, this group was not closely related to the T1 and T3 cells, instead it formed a distantly related sister group. The relationship between T1 and CTC2 is supported by the MNTD statistics on the Bayesian phylogeny.

Neither MNTD nor MPD statistics on the maximum likelihood and Bayesian phylogeny supported the clustering of CTC2 cells. This might suggest that the CTC2 cells are polyphyletic, with their origin in the seeding population before the injection. This is not unlikely, given that the cell lineage used (MDA-MB-231-LM2) is highly metastatic (Minn et al, 2005).

In addition to testing on the best phylogeny, we have integrated the topological uncertainty of the reconstructed phylogenies by performing the phylogenetic clustering tests on the 100 bootstrap replicates from the maximum likelihood analysis and a sample of 1000 trees from the Bayesian posterior tree sample. The distribution of MPD and MNTD *p*-values calculated on each tree were then summarized using mean and 95% confidence interval. The majority of relationships from the best tree was also supported by the tree samples (Supplementary Table S1). This suggests that, while there is high uncertainty in the data and reconstructed topologies, we can reconstruct broad topological patterns with relatively high certainty.

#### 3.1.5. Phylogenetic reconstruction from the SNV data

**FIG. 2.**
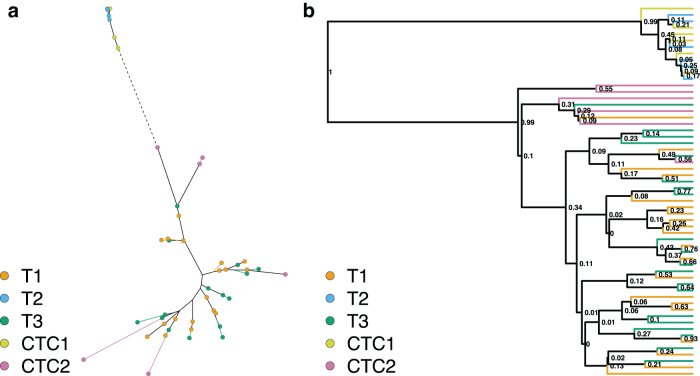
**(a)** Maximum likelihood and **(b)** Bayesian trees reconstructed from the SNV data for the 58 selected cells. Terminal branches are colored according to cell's sample of origin (T1, T2, T3, CTC1, and CTC2). In the maximum likelihood tree, cells are also marked with colored circles and an extremely long branch leading to T2 cells (dashed line) was collapsed. For the Bayesian tree, Bayesian posterior values show the topology uncertainty. SNV, single nucleotide variant.

The maximum likelihood tree reconstructed from the reduced SNV dataset (Fig. 2) displayed similar, but weaker patterns to the one reconstructed from the expression data. The CTC2 cells no longer formed two compact clusters and were dispersed along the tree. Similar to the expression data, the T2 and CTC1 cells were placed together on a long branch, suggesting a long shared evolutionary history. However, unlike the expression data, the T1 and T3 were more interspersed with very short branches. The phylogenetic clustering tests confirm the grouping of all samples (Table 2), except for the CTC2 sample, in addition to the putative relationship between T1 and CTC2, and T2 and CTC1 samples. This reinforces the hypothesis about possible mislabeling between CTC1 and CTC2 samples.

A similar pattern of sample clustering can be observed on the Bayesian phylogeny reconstructed from the same data (Fig. 2b), with T2 and CTC1 cells placed on a distantly related sister branch to all other samples. The T1 and T3 cells are still interspersed, but the CTC2 cells seem to cluster together more closely. Like with the expression analysis, these relationships are stable when the topological uncertainty is taken into account in the phylogenetic clustering tests (Supplementary Table S1).

Additional topological comparison between trees from SNV and expression data can be found in Supplementary Materials S1.

#### 3.1.6. Biological zero or unknown value

To test the assumption if zero expression values should be treated as unknown data rather than biological zeros, that is, no expression of a particular gene, we have reconstructed the phylogenies from the scRNA-seq expression by treating the zeros in the dataset as biological zeros. Data were processed as per the standard methodology to get the alignments, but instead of treating the zeros as an unknown position, they were treated as a category 0, in addition to the five-level ordinal scale. Phylogenies were then reconstructed using both maximum likelihood and Bayesian methods with sample clustering explored using the phylogenetic clustering tests.

**FIG. 3.**
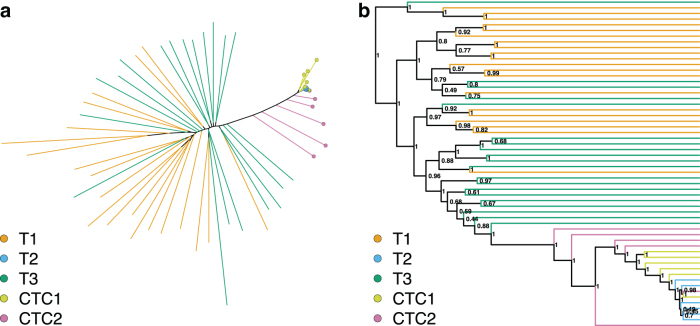
**(a)** Maximum likelihood and **(b)** Bayesian trees reconstructed from the expression data for the 58 selected cells. Terminal branches are colored according to cell's sample of origin (T1, T2, T3, CTC1, and CTC2). In the maximum likelihood tree, the T2, CTC1, and CTC2 samples are also marked with colored circles. For the Bayesian tree, Bayesian posterior values show the topology uncertainty.

In the phylogenies reconstructed from the expression data when zero is treated as a biological zero (Fig. 3), the CTC2 cells did not form a cluster, but clustered closely with the T1 and CTC2 cluster. This cluster was no longer placed as a sister branch to the T1 and T3 cells, but was deeply nested. The T1 and T3 samples were less interspersed than when zero is treated as unknown data. This change in the phylogenetic structure is supported by the phylogenetic clustering tests (Table 3), with T1 and T3 no longer being supported, and instead, the clustering of CTC2 cells is being supported in both the maximum likelihood and Bayesian phylogenies. Likewise, the T1 and CTC2 grouping is not supported, as the CTC2 cells group together with the CTC1 and T2 samples.

**Table 3.** Test of Phylogenetic Clustering for the Expression Data When Zero Expression Level Is Treated as Biological Zero

Groups	Cells	Expression (ML)		Expression (BI)	
		MPD	MNTD	MPD	MNTD
T1	20	1.000	1.000	0.998	0.992
T2	6	^*^0.001	^*^0.001	^*^0.001	^*^0.001
T3	20	0.543	0.994	0.804	0.998
CTC1	6	^*^0.001	^*^0.001	^*^0.001	^*^0.001
CTC2	6	^*^0.004	^*^0.013	^*^0.001	^*^0.011
T1 and CTC1	26	0.992	0.928	0.968	0.560
T2 and CTC2	12	^*^0.001	^*^0.001	^*^0.001	^*^0.001
T1 and CTC2	26	0.997	0.994	0.991	0.888
T2 and CTC1	12	^*^0.001	^*^0.001	^*^0.001	^*^0.001

MPD and MNTD calculated for the ML and BI trees from the expression data, with zeros treated as biological zeros. *p*-Values for MPD and MNTD were calculated for each sample (T1, T2, T3, CTC1, and CTC2) and expected clustering for cells isolated from a single individual (T1 with CTC1, and T2 with CTC2) and to test a possible mislabeling between CTC1 and CTC2 samples (T1 with CTC2, and T2 with CTC1). Significant *p*-values at α=0.05 after correcting for multiple comparisons using the False Discovery Rate method (Benjamini and Hochberg, 1995) are marked with an asterisk.

^*^ Significant support.

These results do not provide a conclusive answer on which assumption should be preferred. Assuming all zeros to be biological zeros will bias the model as many of those might be technical zeros instead. At the same time, the pattern of expression and nonexpression seems to carry information. This information is lost when all zeros are assumed to be technical zeros and thus unknown data. For our datasets, the assumption of zeros as technical zeros and thus unknown data seems to create better agreement in the phylogenetic structure between the expression and SNVs and thus should be preferred. However, our datasets also suffered from unequal data quality issues (Table 1), and under different conditions, assuming zeros as biological zeros might be preferred.

### 3.2. Intestinal neuroendocrine cancer

Cells were labeled according to their sample of origin (primary tumor and metastasis) and their cell type, which was determined by replicating the analysis from Rao et al (2020a). We have derived two subsets from the expression and SNV data for the INC dataset from Rao et al (2020a), a subset with all cell types and a subset with cancer cells only. To do this, cells were labeled according to their sample of origin (primary tumor and metastasis) and their cell type, which was determined by replicating the analysis from Rao et al (2020a). For each subset, 1000 cells with the least amount of missing data were selected, 500 from the primary tumor and 500 from the metastatic sample.

**FIG. 4.**
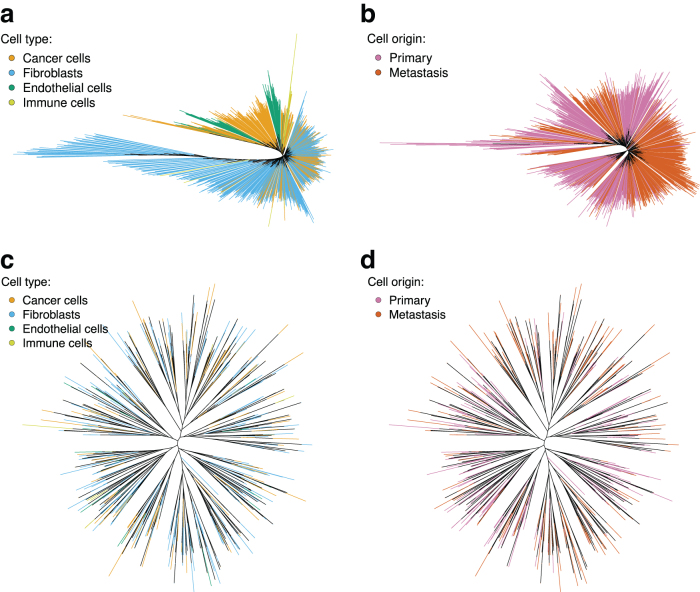
Maximum likelihood trees constructed from the expression and SNV data published by Rao et al (2020a). Terminal branches are colored according to cell's type or sample of origin. In the tree reconstructed from expression data for all cells **(a)**, the vast majority of cancer cells clusters in a single clade. The tree reconstructed from expression data for cancer cells only **(b)** shows a strong clustering of primary and metastatic cells. While the metastatic cells are not clustered in a single clade, multiple metastatic events are biologically plausible. In the trees reconstructed from the SNV data **(c, d)**, primary and metastatic cells, as well as cells of different type, are relatively evenly distributed without any apparent clustering.

However, not all cells found in the expression subsets were found in the SNV data. This is likely due to a different version of the Cellranger software used in this work compared to the Rao et al (2020a). In both derived subsets from the expression data, metastatic cells showed a strong clustering tendency (p=0.001) into several large clades (Fig. 4; Table 4). This suggests a strong phylogenetic relationship with several well-preserved lineages.

**Table 4.** Test of Phylogenetic Clustering on the Maximum Likelihood Trees from Rao et al (2020a)

Data	Groups	Cancer only			All cell types		
		Cells	MPD	MNTD	Cells	MPD	MNTD
Expression	Cancer cells	1000	—	—	355	^*^0.001	^*^0.001
	Fibroblasts	0	—	—	552	1.000	0.989
	Endothelial cells	0	—	—	71	0.860	0.903
	Immune cells	0	—	—	22	0.651	0.565
	Metastasis	500	^*^0.001	^*^0.001	500	^*^0.001	^*^0.001
	Primary	500	1.000	1.000	500	1.000	1.000
SNV	Cancer cells	981	—	—	355	0.362	^*^0.004
	Fibroblasts	0	—	—	552	0.402	0.997
	Endothelial cells	0	—	—	71	0.596	0.785
	Immune cells	0	—	—	22	0.949	0.968
	Metastasis	500	0.907	0.132	500	1.000	^*^0.001
	Primary	481	^*^0.004	0.076	500	^*^0.001	0.095

MPD and MNTD calculated for the phylogeny reconstructed from the dataset containing only cancer cells and from the dataset containing all cell types. *p*-Values for MPD and MNTD were calculated for the sample of origin and cell types where applicable. Significant *p*-values at α=0.05 after correcting for multiple comparisons using the False Discovery Rate method (Benjamini and Hochberg, 1995) are marked with an asterisk.

^*^ Significant support.

In addition, in the derived subset containing all cell types, the cancer cells showed a significant clustering (p=0.001), while other cell types showed the opposite tendency (Table 4). However, the cancer clade contained deeply nested clades of endothelial cells and immune cells. A similar, although significantly weaker pattern of cancer cell clustering can be observed on the trees derived from the SNV data (Table 4). In both subsets derived from the SNV data, the primary cells clustered together, but the pattern was less consistent and confirmed only by one of the two tested statistics.

### 3.3. Gastric cancer

**FIG. 5.**
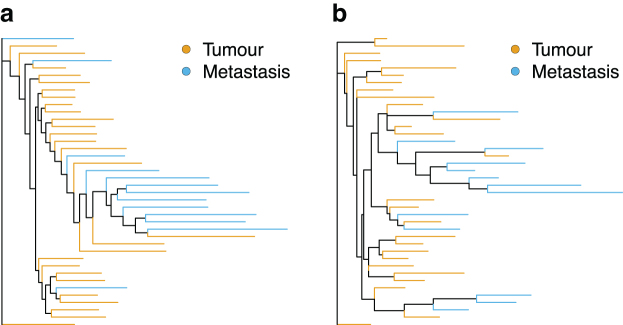
Maximum likelihood trees for the patient G2 constructed from the **(a)** expression and **(b)** SNV data published by Wang et al (2021). Terminal branches are colored according to cell's sample of origin. Only the patient G2 shows a significant clustering signal both on the trees from expression and SNV data. For all trees, see Supplementary Figures S3 and S4.

For both the expression and SNV data from the GC dataset published by Wang et al (2021), only a single patient showed significant clustering of lymph nodes (Fig. 5; Table 5). Poor separation of primary and lymph node cells from the expression levels was pointed out in the original study (Wang et al, 2021). In addition, non-UMI-based methods suffer from an increased error rate through zero-count inflation (Cao et al, 2021) and amplification variability (Townes and Irizarry, 2020). In the absence of a strong phylogenetic signal shared by a large percentage of genes, this additional noise is making a phylogenetic reconstruction difficult, if not impossible. At the same time, the typically higher coverage in the non-UMI-based sequencing compared to the UMI should improve the identification of SNVs and decrease the misspecification error. This might suggest that different strategies for the phylogenetic reconstruction should be applied to UMI- and non-UMI-based sequencing.

**Table 5.** Test of Phylogenetic Clustering on the Maximum Likelihood and Bayesian Trees Calculated from Expression and Single Nucleotide Variant Data Published by Wang et al (2021)

Data	Type	Groups	GC1			GC2			GC3		
			Cells	MPD	MNTD	Cells	MPD	MNTD	Cells	MPD	MNTD
Expression	ML	Primary tumor	19	0.171	0.123	27	^*^0.001	^*^0.001	19	0.829	0.854
		Lymph node	4	0.830	0.869	13	1.000	1.000	12	0.138	0.093
	BI	Primary tumor	19	0.776	0.995	27	0.999	0.953	19	0.218	0.232
		Lymph node	4	0.086	0.035	13	^*^0.002	^*^0.001	12	0.205	0.333
SNV	ML	Primary tumor	19	0.102	0.111	27	^*^0.001	^*^0.001	19	0.552	0.231
		Lymph node	4	0.507	0.092	13	1.000	0.945	12	0.276	0.208
	BI	Primary tumor	19	0.117	0.025	27	^*^0.001	^*^0.014	19	0.531	0.372
		Lymph node	4	0.955	0.935	13	1.000	0.960	12	0.487	0.322

MPD and MNTD calculated for the ML and BI trees reconstructed from the expression and the SNV data for patients GC1, GC2, and GC2. *p*-Values for MPD and MNTD were calculated for the sample of origin. Significant *p*-values at α=0.05 after correcting for multiple comparisons using the False Discovery Rate method (Benjamini and Hochberg, 1995) are marked with an asterisk.

^*^ Significant support.

## 4. DISCUSSION

Phylogenetic methods using scDNA-seq data are becoming increasingly common in tumor evolution studies. scRNA-seq is currently used for studying expression profiles of cancer cells and their behavior. However, while clustering approaches to identify cells with similar expression profiles are common and frequently used, scRNA-seq data are yet to be used in phylogenetic analyses to reconstruct the population history of somatic cells.

To test if the scRNA-seq contains a phylogenetic signal to reliably reconstruct the population history of cancer, we have performed an experiment to produce a known history by infecting immunosuppressed mice with human cancer cells derived from the same population. Then using two different forms of scRNA-seq data, expression values, and SNVs, we reconstructed phylogenies using maximum likelihood and Bayesian phylogenetic methods. By comparing the reconstructed trees to the known population history, we confirmed that scRNA-seq contains a phylogenetic signal to reconstruct the population history of cancer, with both the expression values and SNVs producing a similar phylogenetic pattern.

However, this signal is burdened by uncertainty in both the source data as well as reconstructed phylogeny. Accurate phylogenies might thus need an explicit error model to account for this increased uncertainty (Hicks et al, 2018). Still, by taking this topological uncertainty into account, we can make a conclusion about the structural relationship of individual cells. This highlights that scRNA-seq can be utilized to explore both the physiological behavior of cancer cells and their population history using a single source of data.

While the expression phylogeny can be obtained for virtually no added cost, the choice of normalization method, batch-effect correction, discretization, or the choice between biological and technical zeros can greatly influence the phylogeny. The phylogenies reconstructed from SNV do not suffer from these decisions and SNV-calling pipelines will likely differ only in the number of false negatives and false positives.

Without any specialized phylogenetic or error model for the scRNA-seq data, conventional methods and software tools developed for systematic biology are able to reconstruct population history from these data, potentially at low computational cost. This implies that more accurate inference will be possible when and if specialized models and software are developed, and serious computational resources are employed. For example, computationally more intensive standard nonparametric bootstrap or Bayesian methods on the unfiltered data sets are certainly within the reach of modern computing clusters. This is a future direction for research.

In this work, we tested for phylogenetic signal on three data sets, a new data set consisting of five tumor samples seeded using a population sample, and two previously published data sets consisting of a primary tumor with a paired lymph node or a metastatic sample. The nature of the experiment and the amount of uncertainty in scRNA-seq data barred us from a more detailed exploration of the tree topology as only broad patterns, the phylogenetic clustering of cells according to sample and individual of origin, could be considered. Our clustering analyses show that the phylogenetic trees conform broadly to the expected shapes under different experimental conditions, and thus expression and SNV data can both be used to infer phylogenetic trees from scRNA-seq. Nevertheless, our results also demonstrate that all such trees contain significant uncertainty, so new datasets and methods will be required to extend this work.

The degree to which low and uneven gene expression plays a role in scRNA-seq requires special attention, especially for non-UMI based data sets, as this not only causes a large proportion of missing data but also burdens the known values with a significant error rate. Research should aim at trying to quantify this expression-specific error rate and build specialized models to include the uncertainty about observed data in the phylogenetic reconstruction itself. This could potentially include removing a large proportion of low-coverage data in favor of robust analysis and proper uncertainty estimation of the inferred topology.

The estimation of the topological uncertainty, be it the Bootstrap branch support or the Bayesian posterior clade probabilities, is a staple for phylogenetic analyses. Currently existing methods for the phylogenetic analysis of scDNA-seq, such as SCITE (Jahn et al, 2016), SiFit (Zafar et al, 2017), or SCIΦ (Singer et al, 2018), do not provide this uncertainty estimate. This makes interpretation of the estimated topology difficult because a single topology can only be marginally more accurate than a number of alternative topologies.

Of packages we are aware of, only CellPhy, through its integration in the phylogenetic software RAxML-NG (Kozlov et al, 2019), provides an estimate of topological uncertainty through the bootstrap method. Bayesian methods could be a solution as they provide an uncertainty estimate through the posterior distribution. However, they are significantly more computationally intensive than maximum likelihood methods. Instead, as the size of single-cell data sets will only increase, bootstrap approximations optimized for a large amount of missing data need to be developed to provide a fast and accurate estimate of topological uncertainty.

An aspect of scRNA-seq expression data that was not considered in this study is correlated gene expression (Bageritz et al, 2019; Wang et al, 2004). A single somatic mutation could thus induce a change of expression of multiple genes. This might be problematic, given that phylogenetic methods assume individual sites are independent and this would cause an overestimation of a mutation rate. However, phylogenetic methods are generally rather robust to a wide range of model violations (Huelsenbeck, 1995a,b; Song et al, 2010; Philippe et al, 2011). In addition, by randomly sampling sites, the bootstrap analysis does explore solutions that would arise from this model violation. An investigation of the effect of correlated gene expression on the estimated phylogeny provides an interesting direction for further research.

Multiomic approaches are increasingly popular as they integrate information from multiple biological layers (Bock et al, 2016; Hasin et al, 2017; Nam et al, 2020). While CNVs were ignored in this article, it is possible to detect large-scale CNVs from scRNA-seq data (Gao et al, 2021; Harmanci et al, 2020a,b; Kuipers et al, 2020; Müller et al, 2018). Combined with the SNVs and expression data as analyzed in this article, this enables a multiomic approach using just a single scRNA-seq data source, without the additional cost of DNA sequencing.

## Supplementary Material

Supplemental data
