# Supplementary material for: Testing for Phylogenetic Signal in Single-Cell RNA-Seq Data
Source: J Comput Biol. 2023 Apr 18;30(4):518–37. doi: 10.1089/cmb.2022.0357 (PMC10125402; doi:10.1089/cmb.2022.0357)
Supplement: Supplemental data [file Suppl_Material.docx]

Supplementary Figure 1. Maximum Likelihood trees from the expression data

for 20% (Supplementary Figure 1a), 50% (Supplementary Figure 1b), and 90% (Supplementary Figure 1c) data density. Terminal branches are colored according to cell’s sample of origin (T1, T2, T3, CTC1, CTC2). The T2, CTC1 and CTC2 samples are marked with colored circles.

Supplementary Figure 2. Maximum Likelihood trees from the SNV data for

20% (Supplementary Figure 2a), 50% (Supplementary Figure 2b), and 90% (Supplementary Figure 2c) data density. Terminal branches are colored according to cell’s sample of origin (T1, T2, T3, CTC1, CTC2). The T2, CTC1 and CTC2 samples are marked with colored circles.

Supplementary Figure 3. Maximum Likelihood Bayesian trees constructed from the expression data published by Wang et al. (2021). Terminal branches are colored according to cell’s sample of origin.

Supplementary Figure 4. Maximum Likelihood Bayesian trees constructed from

the SNV data published by Wang et al. (2021). Terminal branches are colored

according to cell’s sample of origin.

**TABLES**

|  |  |  |  | GC1 |  |  | GC2 |  |  | GC3 |  |
| --- | --- | --- | --- | --- | --- | --- | --- | --- | --- | --- | --- |
| Data | Type | Groups | Cells | MPD | MNTD | Cells | MPD | MNTD | Cells | MPD | MNTD |
| Expression | ML | Primary tumor | 19 | 0.171 | 0.123 | 27 | 0.001 | 0.001 | 19 | 0.829 | 0.854 |
|  |  | Lymph node | 4 | 0.830 | 0.869 | 13 | 1.000 | 1.000 | 12 | 0.138 | 0.093 |
|  | BI | Primary tumor | 19 | 0.776 | 0.995 | 27 | 0.999 | 0.953 | 19 | 0.218 | 0.232 |
|  |  | Lymph node | 4 | 0.086 | 0.035 | 13 | 0.002 | 0.001 | 12 | 0.205 | 0.333 |
| SNV | ML | Primary tumor | 19 | 0.102 | 0.111 | 27 | 0.001 | 0.001 | 19 | 0.552 | 0.231 |
|  |  | Lymph node | 4 | 0.507 | 0.092 | 13 | 1.000 | 0.945 | 12 | 0.276 | 0.208 |
|  | BI | Primary tumor | 19 | 0.117 | 0.025 | 27 | 0.001 | 0.014 | 19 | 0.531 | 0.372 |
|  |  | Lymph node | 4 | 0.955 | 0.935 | 13 | 1.000 | 0.960 | 12 | 0.487 | 0.322 |

 significant support

Supplementary Table 1: Test of phylogenetic clustering for the reduced dataset of the 58 selected cells. Mean Pairwise Distance (MPD) and Mean Nearest Taxon Distance (MNTD) calculated for the Maximum Likelihood (ML) and Bayesian (BI) trees from the expression and SNV data. P-values for MPD and MNTD were calculated for each sample (T1, T2, T3, CTC1, CTC2) and expected clustering for cells isolated from a single individual (T1 with CTC1, and T2 with CTC2) and to test a possible mislabeling between CTC1 and CTC2 samples (T1 with CTC2, and T2 with CTC1). P-values were calculated for the sample of 100 bootstrap trees and 1000 posterior trees from the Maximum Likelihood and Bayesian analyses respectively, and this distribution of p-values is summarized with mean and 95% confidence interval. Significant p-values at $\alpha=0.05$ after correcting for multiple comparisons using the False Discovery Rate method (Benjamini and Hochberg, 1995) are marked with an asterisk.

|  |  | Expression (ML) |  | Expression (BI) |  | SNV (ML) |  | SNV (BI) |  |
| --- | --- | --- | --- | --- | --- | --- | --- | --- | --- |
| Groups | Cells | MPD | MNTD | MPD | MNTD | MPD | MNTD | MPD | MNTD |
| T1 | 20 | 0.001 (0.001–0.003) | 0.752 (0.637–0.877) | 0.001 (0.001–0.001) | 0.007 (0.001–0.019) | 0.001 (0.001–0.002) | 0.059 (0.001–0.192) | 0.001 (0.001–0.001) | 0.167 (0.001–0.563) |
| T2 | 6 | 0.001 (0.001–0.001) | 0.001 (0.001–0.001) | 0.002 (0.001–0.006) | 0.004 (0.001–0.017) | 0.017 (0.001–0.001) | 0.032 (0.001–0.005) | 0.003 (0.001–0.001) | 0.005 (0.001–0.007) |
| T3 | 20 | 0.001 (0.001–0.001) | 0.392 (0.280–0.526) | 0.001 (0.001–0.002) | 0.040 (0.002–0.095) | 0.002 (0.001–0.004) | 0.386 (0.021–0.780) | 0.001 (0.001–0.002) | 0.268 (0.009–0.703) |
| CTC1 | 6 | 0.004 (0.001–0.012) | 0.005 (0.001–0.013) | 0.988 (0.971–0.999) | 0.470 (0.280–0.621) | 0.011 (0.001–0.002) | 0.024 (0.001–0.015) | 0.002 (0.001–0.002) | 0.007 (0.001–0.023) |
| CTC2 | 6 | 1.000 (1.000–1.000) | 0.987 (0.937–1.000) | 0.701 (0.648–0.747) | 0.946 (0.845–1.000) | 0.237 (0.161–0.347) | 0.491 (0.230–0.727) | 0.175 (0.083–0.247) | 0.433 (0.183–0.628) |
| T1 & CTC1 | 26 | 0.051 (0.022–0.082) | 0.427 (0.273–0.641) | 0.146 (0.110–0.180) | 0.281 (0.049–0.499) | 0.426 (0.245–0.557) | 0.084 (0.001–0.257) | 0.393 (0.262–0.521) | 0.225 (0.001–0.748) |
| T2 & CTC2 | 12 | 0.995 (0.986–1.000) | 0.092 (0.002–0.321) | 0.949 (0.912–0.979) | 0.127 (0.004–0.304) | 0.950 (0.663–1.000) | 0.539 (0.119–0.854) | 0.938 (0.859–0.996) | 0.379 (0.077–0.680) |
| T1 & CTC2 | 26 | 0.574 (0.360–0.716) | 1.000 (0.998–1.000) | 0.005 (0.002–0.009) | 0.889 (0.741–0.988) | 0.001 (0.001–0.002) | 0.450 (0.062–0.826) | 0.001 (0.001–0.003) | 0.465 (0.059–0.896) |
| T2 & CTC1 | 12 | 0.001 (0.001–0.001) | 0.001 (0.001–0.001) | 0.654 (0.556–0.758) | 0.053 (0.001–0.173) | 0.004 (0.001–0.027) | 0.060 (0.001–0.723) | 0.001 (0.001–0.001) | 0.005 (0.001–0.003) |

 significant support

(a) Pairwise nMSID

|  | ML |  | BI |  |
| --- | --- | --- | --- | --- |
|  | mean | sd | mean | sd |
| SNV | 0.33 | 0.04 | 0.22 | 0.04 |
| Expr | 0.13 | 0.03 | 0.08 | 0.03 |
| Expr0 | 0.09 | 0.02 | 0.01 | 0.01 |

(b) nMSID distance to tree sample

|  | SNV | Expr | Expr0 |
| --- | --- | --- | --- |
| SNV | 0.00 | 0.59 | 0.56 |
| Expr | 0.59 | 0.00 | 0.71 |
| Expr0 | 0.56 | 0.71 | 0.00 |

(c) Pairwise RNNI distances

Supplementary Table 2. Comparison of distances between trees made from SNV and expression data from the BCX dataset. Table 2a shows pairwise distances between the best trees from SNV, expression values, and expression values where not expressed genes are considered true biological zeroes (Expr0), from both Maximum Likelihood and Bayesian Inference. Table 2b summarizes the distance between these trees and their respective bootstrap or posterior tree samples. Table 2c shows pairwise RNNI distances between the best trees from SNV, expression values, and expression values where not expressed genes are considered true biological zeroes from Bayesian Inference

Supplementary Table 3. Test of phylogenetic clustering for the datasets filtered to 20%, 50% and 90% data density. Mean Pairwise Distance (MPD) and Mean Nearest Taxon Distance (MNTD) calculated for the Maximum Likelihood (ML) and Bayesian (BI) trees from the expression and SNV data. P-values for MPD and MNTD were calculated for each sample (T1, T2, T3, CTC1, CTC2) and exprected clustering for cells isolated from a single individual (T1 with CTC1, and T2 with CTC2) and to test a possible mislabeling between CTC1 and CTC2 samples (T1 with CTC2, and T2 with CTC1). Significant p-values at *α* = 0.05 after correcting for multiple comparisons using the False Discovery Rate method (Benjamini and Hochberg, 1995) are marked with an asterisk.

|  |  | 20% data density |  |  |  | 50% data density |  | 90% data density |  |  |
| --- | --- | --- | --- | --- | --- | --- | --- | --- | --- | --- |
| Data | Groups | Cells | MPD | MNTD | Cells | MPD | MNTD | Cells | MPD | MNTD |
| Expression | T1 | 701 | 1.000 | 1.000 | 688 | 1.000 | 0.996 | 329 | 0.910 | 0.089 |
|  | T2 | 11 | *0.001 | *0.001 | 0 |  |  | 0 |  |  |
|  | T3 | 806 | 0.126 | 1.000 | 758 | *0.001 | *0.001 | 262 | 0.015 | *0.002 |
|  | CTC1 | 58 | *0.001 | *0.001 | 3 | 0.156 | 0.099 | 0 |  |  |
|  | CTC2 | 51 | *0.001 | *0.001 | 5 | 1.000 | 1.000 | 2 | 1.000 | 1.000 |
|  | T1 & CTC1 | 759 | 0.992 | 0.999 | 691 | 1.000 | 0.998 | 329 | 0.910 | 0.089 |
|  | T2 & CTC2 | 62 | *0.001 | *0.001 | 5 | 1.000 | 1.000 | 2 | 1.000 | 1.000 |
|  | T1 & CTC2 | 752 | 1.000 | 1.000 | 693 | 1.000 | 1.000 | 331 | 0.972 | 0.148 |
|  | T2 & CTC1 | 69 | *0.001 | *0.001 | 3 | 0.156 | 0.099 | 0 |  |  |
| SNV | T1 | 352 | *0.001 | *0.001 | 55 | 0.066 | 0.276 | 13 | 0.221 | 0.200 |
|  | T2 | 0 |  |  | 0 |  |  | 0 |  |  |
|  | T3 | 514 | 1.000 | 0.999 | 196 | 0.795 | 0.571 | 47 | 0.801 | 0.736 |
|  | CTC1 | 0 |  |  | 0 |  |  | 0 |  |  |
|  | CTC2 | 4 | 0.520. | 0.557 | 3 | 0.892 | 0.695 | 0 |  |  |
|  | T1 & CTC1 | 352 | *0.001 | *0.001 | 55 | 0.066 | 0.276 | 13 | 0.221 | 0.200 |
|  | T2 & CTC2 | 4 | 0.520 | 0.557 | 3 | 0.892 | 0.695 | 0 |  |  |
|  | T1 & CTC2 | 356 | *0.001 | *0.001 | 58 | 0.152 | 0.558 | 13 | 0.221 | 0.200 |
|  | T2 & CTC1 | 0 |  |  | 0 |  |  | 0 |  |  |

* significant support

Supplementary Table 4. Test of phylogenetic clustering on the Maximum Likelihood boostrap trees reconstructed from the expression data published by Rao et al. (2020a). Mean Pairwise Distance (MPD) and Mean Nearest Taxon Distance (MNTD) calculated for the Maximum Likelihood boostrap trees reconstructed from the expression dataset containing only cancer cells and from the dataset containing all cell types. P-values were calculated for the sample of 100 bootstrap trees and 1000 posterior trees from the Maximum Likelihood and Bayesian analyses respectively, and this distribution of p-values is summarized with mean and 95% confidence interval. Significant p-values at *α* = 0.05 after correcting for multiple comparisons using the False Discovery Rate method (Benjamini and Hochberg, 1995) are marked with an asterisk.

The bootstrap analysis for the SNV dataset failed to finish after 30 days and was terminated. For this reason, the phylogenetic clustering tests for the bootstrap trees from the SNV data are omitted.

|  |  |  | Cancer only | |  |  |  | All cell types |  |  |
| --- | --- | --- | --- | --- | --- | --- | --- | --- | --- | --- |
| Groups | Cells |  | MPD |  | MNTD | Cells |  | MPD |  | MNTD |
| Cancer cells | 1000 |  | – |  | – | 355 | ^*^0.001 | (0.001–0.001) | ^*^0.001 | (0.001–0.001) |
| Fibroblasts | 0 |  | – |  | – | 552 | 1.000 | (0.999–1.000) | 0.986 | (0.967–1.000) |
| Endothelial cells | 0 |  | – |  | – | 71 | 0.797 | (0.679–0.914) | 0.901 | (0.811–0.977) |
| Immune cells | 0 |  | – |  | – | 22 | 0.734 | (0.629–0.828) | 0.616 | (0.411–0.783) |
| Metastasis | 500 | ^*^0.001 | (0.001–0.001) | ^*^0.001 | (0.001–0.001) | 500 | ^*^0.001 | (0.001–0.001) | ^*^0.001 | (0.001–0.001) |
| Primary | 500 | 1.000 | (1.000–1.000) | 0.998 | (0.992–1.000) | 500 | 1.000 | (1.000–1.000) | 1.000 | (1.000–1.000) |

* significant support

Supplementary Table 5. Comparison of trees reconstructed from the SNV and expression data published by Rao et al. (2020a) using nMSID. The nMSID was calculated between tree reconstructed from SNV and a tree reconstructed for expression data and compared to a expected random nMSID for trees with the same amount of taxa. For both subset (All cells and Cancer cells only), the calculated nMSID (0.70 and 0.69) were larger than expected for random trees. The information in SNV and expression values seems to be diverging.

|  | cells | nMSID | E(nMSID) |
| --- | --- | --- | --- |
| All cells | 1000 | 0.70 | 0.63 |
| Cancer only | 981 | 0.69 | 0.62 |

Supplementary Table 6. Test of phylogenetic clustering on the sample of Maximum Likelihood and Bayesian trees calculated from expression and SNV data published by Wang et al. (2021). Mean Pairwise Distance (MPD) and Mean Nearest Taxon Distance (MNTD) calculated for the Maximum Likelihood boostrap trees and Bayesian posterior sample of trees reconstructed from the expression and the SNV data for patients GC1, GC2 and GC2. P-values were calculated for the sample of 100 bootstrap trees and 1000 posterior trees from the Maximum Likelihood and Bayesian analyses respectively, and this distribution of p-values is summarized with mean and 95% confidence interval. Significant p-values at *α* = 0.05 after correcting for multiple comparisons using the False Discovery Rate method (Benjamini and Hochberg, 1995) are marked with an asterisk.

|  |  |  |  | GC1 |  |  | GC2 |  |  | GC3 |  |
| --- | --- | --- | --- | --- | --- | --- | --- | --- | --- | --- | --- |
| Data | Type | Groups | Cells | MPD | MNTD | Cells | MPD | MNTD | Cells | MPD | MNTD |
| Expression | ML | Primary tumor | 19 | 0.181 (0.157–0.199) | 0.146 (0.092–0.189) | 27 | *0.001 (0.001–0.001) | *0.001 (0.001–0.001) | 19 | 0.825 (0.731–0.882) | 0.869 (0.774–0.926) |
|  |  | Lymph node | 4 | 0.816 (0.788–0.848) | 0.877 (0.723–0.979) | 13 | 0.999 (0.991–1.000) | 1.000 (0.999–1.000) | 12 | 0.143 (0.075–0.191) | 0.108 (0.041–0.187) |
|  | BI | Primary tumor | 19 | 0.661 (0.122–1.000) | 0.793 (0.402–1.000) | 27 | 0.932 (0.692–1.000) | 0.866 (0.569–1.000) | 19 | 0.394 (0.018–0.894) | 0.369 (0.002–0.814) |
|  |  | Lymph node | 4 | 0.242 (0.001–0.682) | 0.184 (0.001–0.582) | 13 | 0.033 (0.001–0.153) | 0.022 (0.001–0.101) | 12 | 0.415 (0.001–0.910) | 0.424 (0.010–0.901) |
| SNV | ML | Primary tumor | 19 | 0.198 (0.002–0.528) | 0.274 (0.005–0.682) | 27 | *0.001 (0.001–0.001) | *0.019 (0.001–0.113) | 19 | 0.594 (0.368–0.864) | 0.441 (0.074–0.900) |
|  |  | Lymph node | 4 | 0.710 (0.225–0.999) | 0.620 (0.132–0.995) | 13 | 1.000 (1.000–1.000) | 0.935 (0.598–1.000) | 12 | 0.383 (0.087–0.658) | 0.425 (0.073–0.806) |
|  | BI | Primary tumor | 19 | 0.207 (0.001–0.544) | 0.127 (0.001–0.470) | 27 | *0.001 (0.001–0.001) | 0.061 (0.001–0.191) | 19 | 0.530 (0.319–0.752) | 0.506 (0.154–0.901) |
|  |  | Lymph node | 4 | 0.762 (0.333–0.999) | 0.761 (0.342–0.999) | 13 | 1.000 (0.998–1.000) | 0.814 (0.421–0.999) | 12 | 0.470 (0.268–0.677) | 0.362 (0.031–0.701) |

* significant support

|  |  | ML |  | BI |  |
| --- | --- | --- | --- | --- | --- |
|  |  | SNV | Expr | SNV | Expr |
| ML | SNV | 0.00 | 0.56 | 0.25 | 0.56 |
|  | Expr | 0.56 | 0.00 | 0.53 | 0.52 |
| BI | SNV | 0.25 | 0.53 | 0.00 | 0.53 |
|  | Expr | 0.56 | 0.52 | 0.53 | 0.00 |

E(nMSID) for 23 taxa is 0.51

|  |  | ML |  | BI |  |
| --- | --- | --- | --- | --- | --- |
|  |  | SNV | Expr | SNV | Expr |
| ML | SNV | 0.00 | 0.53 | 0.47 | 0.57 |
|  | Expr | 0.53 | 0.00 | 0.41 | 0.56 |
| BI | SNV | 0.47 | 0.41 | 0.00 | 0.56 |
|  | Expr | 0.57 | 0.56 | 0.56 | 0.00 |

E(nMSID) for 40 taxa is 0.54

(a) nMSID(GC1)

|  |  | ML |  | BI |  |
| --- | --- | --- | --- | --- | --- |
|  |  | SNV | Expr | SNV | Expr |
| ML | SNV | 0.00 | 0.56 | 0.39 | 0.57 |
|  | Expr | 0.56 | 0.00 | 0.51 | 0.51 |
| BI | SNV | 0.39 | 0.51 | 0.00 | 0.53 |
|  | Expr | 0.57 | 0.51 | 0.53 | 0.00 |

E(nMSID) for 31 taxa is 0.53

|  | RNNI |
| --- | --- |
| GC1 | 0.82 |
| GC2 | 0.77 |
| GC3 | 0.68 |

(d) Pairwise RNNI distances

Supplementary Table 7. Comparison of distances between trees made from SNV and expression data from the GC dataset. Tables 7a, 7b and 7c show pairwise nMSID between the best trees from SNV and expression values from Maximum Likelihood and Bayesian Inference for patients GC1, GC2 and GC3 respectively. Table 7d shows pairwise RNNI distances between SNV and expression values for Bayesian trees for all three patients. Many of the nMSID for all three patients are close to the expected random nMSID (E(nMSID)). This suggests that there is a significant difficulty in reliably estimating a correct topology, regardless of the type of data (SNV or expression).
